# Is Upregulation of BCL2 a Determinant of Tumor Development Driven by Inactivation of CDH1/E-Cadherin?

**DOI:** 10.1371/journal.pone.0073062

**Published:** 2013-08-30

**Authors:** Inga Karch, Elisa Schipper, Henriette Christgen, Hans Kreipe, Ulrich Lehmann, Matthias Christgen

**Affiliations:** Institute of Pathology, Medizinische Hochschule Hannover, Hannover, Germany; National Cancer Center, Japan

## Abstract

Inactivation of *CDH1*, encoding E-cadherin, promotes cancer initiation and progression. According to a newly proposed molecular mechanism, loss of E-cadherin triggers an upregulation of the anti-apoptotic oncoprotein BCL2. Conversely, reconstitution of E-cadherin counteracts overexpression of BCL2. This reciprocal regulation is thought to be critical for early tumor development. We determined the relevance of this new concept in human infiltrating lobular breast cancer (ILBC), the prime tumor entity associated with *CDH1* inactivation. BCL2 expression was examined in human ILBC cell lines (IPH-926, MDA-MB-134, SUM-44) harboring deleterious *CDH1* mutations. To test for an intact regulatory axis between E-cadherin and BCL2, wild-type E-cadherin was reconstituted in ILBC cells by ectopic expression. Moreover, BCL2 and E-cadherin were evaluated in primary invasive breast cancers and in synchronous lobular carcinomas *in situ* (LCIS). MDA-MB-134 and IPH-926 showed little or no BCL2 expression, while SUM-44 ILBC cells were BCL2-positive. Reconstitution of E-cadherin failed to impact on BCL2 expression in all cell lines tested. Primary ILBCs were almost uniformly E-cadherin-negative (97%) and were frequently BCL2-negative (46%). When compared with an appropriate control group, ILBCs showed a trend towards an increased frequency of BCL2-negative cases (*P = *0.064). In terminal duct-lobular units affected by LCIS, the E-cadherin-negative neoplastic component showed a similar or a reduced BCL2-immunoreactivity, when compared with the adjacent epithelium. In conclusion, upregulation of BCL2 is not involved in lobular breast carcinogenesis and is unlikely to represent an important determinant of tumor development driven by *CDH1* inactivation.

## Introduction

E-cadherin is a transmembrane glycoprotein that mediates calcium-dependent cell-cell adhesion in epithelial tissues. Loss of E-cadherin has mainly been implicated in cancer progression [1]. Experimental animal models have shown that loss of E-cadherin induces epithelial-mesenchymal transition (EMT) and thereby promotes metastatic dissemination [2]. However, E-cadherin has a much wider implication in human cancer biology. The *CDH1* gene, which encodes for E-cadherin, functions as a tumor suppressor gene and *CDH1* germline mutations are associated with a hereditary tumor syndrome [3], [4]. Thus, loss of E-cadherin can initiate tumor development. The molecular mechanisms that drive tumor formation following *CDH1* inactivation are controversial and may include aberrant activation of the WNT signaling pathway through β-catenin, induction of anoikis-resistance through p120-catenin and cytoplasmic mislocalization of Kaiso, a transcriptional modulator [5], [6], [7]. A new molecular mechanism involving the BCL2 oncoprotein has recently been proposed by Ferreira and colleagues [8]. According to this new concept, loss of E-cadherin triggers an upregulation of the anti-apoptotic oncoprotein BCL2, a process mediated by upregulation of Notch-1 expression and activity, and thereby increases cell survival [8]. Conversely, reconstitution of E-cadherin has been reported to counteract overexpression of BCL2 [8]. This reciprocal regulation may be a critical determinant of early tumor development following *CDH1* inactivation or loss of E-cadherin expression [8].

Infiltrating lobular breast cancer (ILBC) is a special breast cancer subtype and accounts for 5 - 15% percent of all mammary carcinomas [9]. ILBCs consist of small, discohesive epithelial cells, which are individually dispersed or arranged in single file linear cords [10]. From a clinical point of view, ILBC is an indolent, hormone-responsive and slowly-progressive malignancy [11]. From a cell biology perspective, ILBC is the most important model disease for studying carcinogenesis driven by *CDH1* inactivation [2], [12], [13], [14], [15], [16], [17]. In fact, ILBCs are almost uniformly E-cadherin-negative and harbor deleterious *CDH1* mutations [12], [13], [16], [17]. Loss of E-cadherin is otherwise rare in breast cancer. Complete absence of E-cadherin or a full-blown EMT phenotype are encountered in less than 5% of infiltrating ductal breast cancers (IDBC), which account for the vast majority of all mammary carcinomas [18], [19]. Of note, ILBCs can arise from a non-obligate intraepithelial precursor lesion termed lobular carcinoma *in situ* (LCIS) [10]. In LCIS, the tumor cells are confined to terminal duct-lobular units (TDLUs) and have not yet infiltrated the basement membrane [10]. E-cadherin is already lost in the neoplastic cellular component of TDLUs affected by LCIS [15]. Hence, LCIS provides the opportunity for studying gene expression alterations associated with the inactivation of E-cadherin in comparison to the immediately adjacent, non-neoplastic epithelium [15].

Using human ILBC cell lines, primary tumors and pre-invasive lesions as a model, the present study aimed to determine the relevance of the newly proposed relationship between E-cadherin and BCL2 for tumor development driven by *CDH1* inactivation.

## Materials and Methods

### Cell Culture

The human breast cancer cell lines MDA-MB-134 and IPH-926 have been described previously [20], [21], [22], [23], [24], [25]. BT-549 and MDA-MB-435/M14 cells were obtained by American Type Culture Collection (ATCC, Manassas, VA, U.S.A.). SUM-44-PE cells were kindly provided by D. Derksen [26]. Cell line characteristics are summarized below **(** **Table 1** **)**. All cell lines were authenticated by short tandem repeat (STR) profiling. MDA-MB-134, IPH-926 and SUM-44-PE cells were additionally authenticated by detection of their unique, homozygous *CDH1* mutations **(** **Table 1** **)** [5], [20], [24]. All cell lines were routinely cultured in RPMI-1640 medium supplemented with 10% FCS, 10 µg/ml bovine insulin, 2.5 g/l glucose, 1 mM sodium pyruvate, 2 mM glutamine, and 10 mM HEPES, in a water-saturated atmosphere containing 5% CO_2_ at 37.5°C.

**Table 1:** Tumor cell line characteristics.

			*CDH1* status, cell line			CDH1 status,	
cell line	origin	established	nucleotide	meth.	protein	primary	ref.
**IPH-926**	ILBC^1^	2006	241ins4 (homo)	neg	neg	241ins4 (homo)	[20]–[22]
**MDA-MB-134**	ILBC^2^	1973	688del145 (homo)	neg	neg	na	[23]–[25]
**SUM-44-PE**	ILBC^2^	1993	1269delT (homo)	neg	neg	na	[5]; [26]
**MDA-MB-435/M14**	melanoma	1976	wt	pos	neg	na	[37]–[39]
**BT-549**	PABC	1978	wt	pos	neg	na	[36]; [37]

1 origin from ILBC proven by genetic comparison with the corresponding primary ILBC.

2 origin from ILBC proposed *ex post* based on molecular features, corresponding primary tumor remained uncharacterized ILBC; infiltrating lobular breast cancer, PABC; papillary breast cancer, homo; homozygous, meth.; aberrant methylation of the *CDH1* promoter, neg; negative, pos; positive, na; not assessed.

### Reconstitution of E-cadherin

Cells were transiently transfected either with vector pEGFP-N2 (Clontech Laboratories, Mountain View, CA, U.S.A.) or with the p-wtEcad-EGFP-N2 expression construct encoding for full-length wild-type human E-cadherin fused to the cDNA sequence of EGFP, which was kindly provided by B. Luber [27]. Transfection reactions were carried out using Lipofectamine™ 2000 (Invitrogen, Darmstadt, Germany) according to manufacturer’s recommendations.

### Fluorescent Imaging

Cells were grown on LAB-TEK-II chamber slides (Thermo Fisher Scientific, Waltham, MA, U.S.A.) for 24 h after the transfection. Next, cells were fixed in 4% paraformaldehyde, and were incubated with a mouse monoclonal anti-p120-catenin antibody (clone 98, 2.5 ng/µl, BD Transduction Laboratories, Heidelberg, Germany) or with a mouse monoclonal anti-Kaiso antibody (clone 6F, 2 ng/µl, Merck Millipore, Darmstadt, Germany). Then, cells were incubated with a secondary goat anti-mouse antibody labeled with Alexa Fluor 647 (10 ng/µl, Invitrogen, Darmstadt, Germany). Finally, cells were counterstained with DAPI (2 µg/ml, Sigma-Aldrich, St. Louis, MI, U.S.A) and were mounted in ProLong Gold cover medium (Invitrogen, Darmstadt, Germany). Fluorescent imaging was performed with a Leica Inverted-2 confocal laser scanning microscope (Leica Microsystems, Wetzlar, Germany).

### Cell Sorting

Pre-analytical enrichment of EGFP- or Ecad-EGFP-positive cells (minimum 70% purity) was carried out by FACS sort using a MoFlo cell sorter (Cytomation, Fort Collins, CO, U.S.A.) [28]. Subsequent expression analyses and cell aggregation assays were performed immediately after the FACS sort, without any interim expansion in cell culture.

### Cell Aggregation Assay

Single cell sorted EGFP- or Ecad-EGFP-positive cell preparations were incubated in either normal growth medium or medium supplemented with 2.5 mM EDTA for 45 minutes at 37°C. Relative cell aggregation was assessed by flow cytometry-based detection of aggregates on a MoFlo cytometer. Relative cell cohesion was expressed as a cell aggregation index (CAI), representing the percentage of aggregated cells in the test condition (Ecad-EGFP, with or without EDTA) divided by the percentage of aggregated cells in the control condition (EGFP), as described previously [29], [30].

### Western Blot

Cells were lysed in RIPA buffer and 20 µg total cellular protein were separated by SDS-PAGE and were transferred to nitrocellulose membranes. Membranes were probed with anti-E-cadherin (clone 36, BD Transduction Laboratories, Heidelberg, Germany), anti-BCL2 (clone 124, Dako, Glostrup, Denmark), anti-Notch-1 (clone c20, Santa Cruz Biotechnology, Heidelberg, Germany) and anti–β-actin antibodies (clone AC15, Acris, Hiddenhausen, Germany), as described previously [31]. 293T cells transfected with a Notch-1 expression plasmid (sc-110326, Santa Cruz Biotechnology) served as a positive control for Notch-1.

### Tumor Specimen and Tissue Microarrays

Formalin-fixed paraffin-embedded (FFPE) human primary invasive breast cancer specimens (n = 172) were retrieved from the tissue archive of the Institute of Pathology of the Hannover Medical School according to the guidelines of the local ethics committee ("Ethik-Kommission der Medizinischen Hochschule Hannover", head: Prof. Dr. Tröger). This study was exempted from review from the local ethics committee since the specimens used in this study were left-over samples from diagnostic procedures and thereby retrieved retrospectively. All specimens were made anonymous and waived the need for consent. They were compiled on tissue microarrays (TMAs), as described previously [32], [33]. Tumor characteristics are summarized below **(** **Table 2** **)**. Tumor characteristics of the subset of estrogen receptor (ER)-positive cases are provided in Table S2. From the primary ILBCs represented on the TMAs, we selected n = 11 cases with synchronous LCIS in separate FFPE tissue blocks for analysis of pre-invasive tumor cells.

**Table 2:** Characteristics of primary tumors.

	number of cases	percent
***cases***	172	100
***age***		
>60	72	42
≤60	100	58
***histological type***		
ILBC	37	22
IDBC	135	78
***pT status***		
pT1/pT2	149	86
pT3/pT4	22	13
pTx	1	1
***pN status***		
pN0	94	55
pN1+	57	33
pNx	21	12
***histological grade***		
G1	15	9
G2	97	56
G3	60	35
***estrogen receptor***		
Positive	139	81
negative	33	19
***progesterone receptor***		
Positive	100	58
negative	72	42
***c-erbB2 expression***		
0, 1+	161	94
2+	3	2
3+	8	4
***E-cadherin (in ILBC)***		
Positive	1	3
negative	36	97
***E-cadherin (in IDBC)***		
Positive	130	96
negative	5	4
***Ki67 LI***		
≤10	30	17
>10, <24	89	52
≥25	53	31

### Immunohistochemistry

Four micrometer sections of TMAs or FFPE breast tissue were mounted on Superfrost slides (Thermo Fisher Scientific). Slides were deparaffinized and rehydrated conventionally. Staining was performed on a Benchmark Ultra (Ventana, Tucson, AZ, U.S.A.) automated stainer using the CC1 mild protocol for antigen retrieval, monoclonal anti-E-cadherin (clone 4A2C7, Invitrogen) or anti-BCL2 (clone 124, Dako) antibodies and the ultraView DAB kit for signal detection. Evaluation of BCL2 immunoreactivity was carried out using an immunoreactivity score (IRS) as described by Remmele and Stegener [34]. Tumors with an IRS≤2 were considered as BCL2-negative, whereas those with an IRS≥3 were considered as BCL2-positive. The same IRS score was implemented for evaluation of BCL2 expression and LCIS and at least three TDLU were considered per individual case. For E-cadherin, only cases with complete absence of any membranous E-cadherin immunoreactivity were considered as E-cadherin-negative [33]. Detection and evaluation of estrogen receptor (ER), progesterone receptor (PR), c-erbB2 and Ki67 expression were performed as described previously [33]. Ki67 labeling index cutoffs for definition of low, intermediate and high proliferation were 10 and 25% [33]. Statistical analyses were performed with GraphPads Prism software. Fisher’s exact test or the Chi square test for trends were used to assess associations between clinicopathological variables and the BCL2-status. *P* values <0.05 were considered statistically significant.

## Results

### Reconstitution of E-cadherin Fails to Impact on BCL2 in Human ILBC Cells *in vitro*

According to a newly proposed molecular mechanism, loss of E-cadherin triggers an upregulation of the oncoprotein BCL2 [8]. Conversely, reconstitution of E-cadherin has been reported to counteract overexpression of BCL2 [8]. We sought to study this reciprocal regulation in cell lines derived from ILBC, a tumor entity associated with *CDH1* inactivation. Few ILBC cell lines have been established so far [35]. For our analyses, we selected three cell lines, which are of proven (IPH-926), or proposed (MDA-MB-134 and SUM-44-PE) origin from ILBC and harbor deleterious *CDH1* mutations **(** **Table 1** **)**. We also included BT-549 cells, which were derived from a papillary carcinoma of the breast and display an EMT phenotype *in vitro* [36], [37]. Moreover, we included MDA-MB-435 cells, which were formerly known as breast cancer cells, but were derived from the M14 melanoma cell line instead [38], [39]. MDA-MB-435/M14 was included because this cell line had been used in the original publication describing the reciprocal regulation of E-cadherin and BCL2 [8].

**Figure 1:**
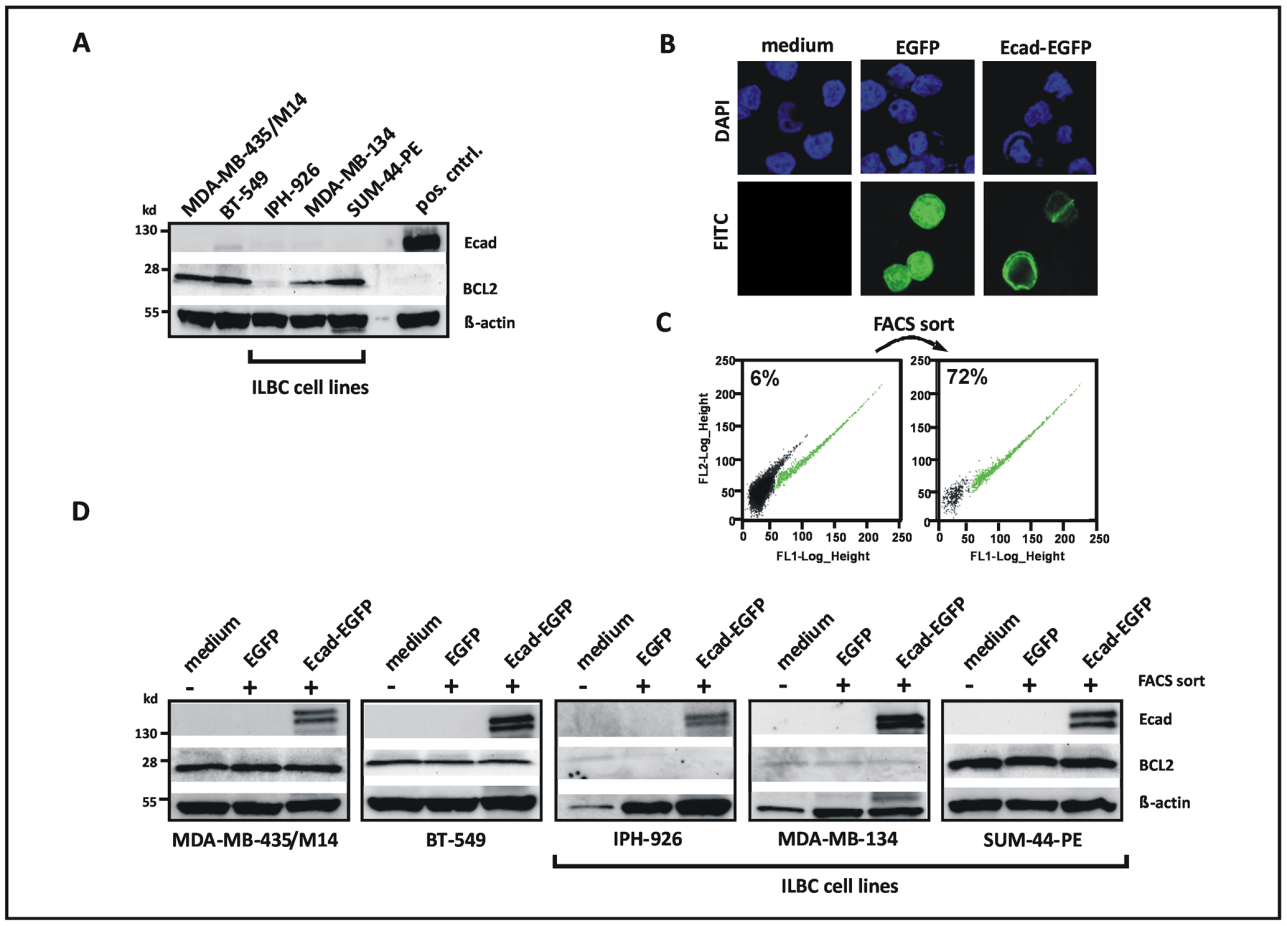
Reconstitution of E-cadherin fails to impact on BCL2 expression in human ILBC cells. (**A**) Analysis of E-cadherin (Ecad) and BCL2 protein expression by Western Blot. Pos cntrl; positive control for E-cadherin. (**B**) Fluorescent imaging of cells subjected to ectopic expression of EGFP or EGFP-tagged E-cadherin (Ecad-EGFP) by transient transfection. Representative photomicrographs show the IPH-926 ILBC cell line. Note the membranous localization of Ecad-EGFP. (**C**) Pre-analytical enrichment of EGFP- or Ecad-EGFP-positive cells by FACS sort. Cells within the EGFP-positive gate are colored in green. Representative dot blots show IPH-926 ILBC cells transfected with the Ecad-EGFP expression construct. (**D**) Analysis of E-cadherin and BCL2 protein expression by Western blot. Cells were subjected to ectopic expression of EGFP or Ecad-EGFP for 24 h and subsequent pre-analytical enrichment of EGFP-positive cells by FACS sort (minimum 70% purity). Untreated cells were included as controls. Ecad-EGFP presented as a double band of approximately 150 kd, as reported previously [27]. Similar results were obtained 48 h after transfection (not shown).

Western blot analysis confirmed lack of E-cadherin in all cell lines studied **(** **Figure 1A** **)**. Interestingly, MDA-MB-134 and IPH-926 ILBC cells showed little or no BCL2 expression, while SUM-44-PE, BT-549 and MDA-MB-435/M14 were BCL2-positive **(** **Figure 1A** **)**. Increased expression and activation of the Notch receptor family member Notch-1 has been proposed to mediate the upregulation of BCL2 in response to inactivation of E-cadherin [8]. However, all E-cadherin-deficient cells tested also lacked detectable Notch-1 expression, regardless of their BCL2 expression status **(Figure S1)**. To test whether reconstitution of E-cadherin downregulates BCL2, as proposed previously [8], cells were transiently transfected with expression plasmids encoding for EGFP-tagged full-length wild-type E-cadherin (Ecad-EGFP) or EGFP alone **(** **Figure 1B–D** **)**. To compensate for limited transfection efficiency, positive cells were enriched by FACS sort **(** **Figure 1C** **)**. Subsequent expression analyses were performed immediately after the FACS sort, without any interim expansion in cell culture. However, reconstitution of E-cadherin failed to impact on BCL2 expression in all cell lines tested, including BCL2-positive MDA-MB-435/M14 and SUM-44-PE ILBC cells **(** **Figure 1D** **)**.

**Figure 2:**
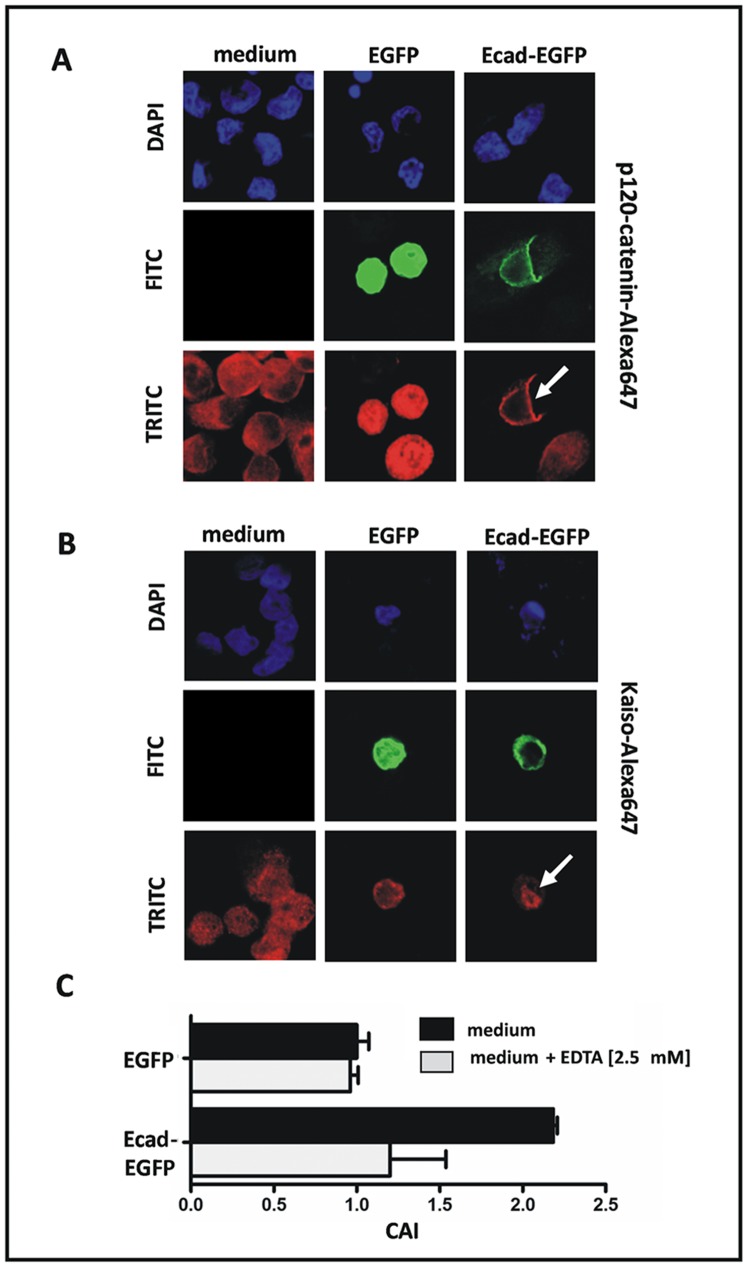
Validation of the functional activity of the E-cadherin construct. (**A**) Fluorescent imaging of cells subjected to ectopic expression of EGFP- or Ecad-EGFP showing the relocation of p120-catenin to the cell membrane in Ecad-EGFP-positive cells. (**B**) Fluorescent imaging showing the relocation of Kaiso to the nucleus. Representative photomicrographs were taken from experiments with the E-cadherin-deficient IPH-926 ILBC cell line. (**C**) Increased calcium-dependent cell-cell adhesion in E-cadherin-deficient IPH-926 ILBC cells transiently transfected Ecad-EGFP. The cell aggregation index (CAI) was determined by FACS analysis as described in the “Material and Methods” section.

This prompted us to test the functionality of the E-cadherin construct. The E-cadherin binding protein p120-catenin shows an aberrant cytoplasmic localization and triggers anoikis resistance in E-cadherin-deficient tumor cells [6]. Besides that, cytoplasmic p120-catenin sequesters the nuclear transcriptional modulator Kaiso, which thus exhibits a cytoplasmic mislocalization, once E-cadherin is lost [7]. Hence, we utilized the subcellular localization of p120-catenin and Kaiso, as detected by immunofluorescence, as a read-out system for the functionality of the E-cadherin construct. E-cadherin-deficient cells transfected with Ecad-EGFP showed a relocation of p120-catenin to the cell membrane **(** **Figure 2A** **)** and a relocation of Kaiso to the nucleus, arguing for the intact functionality of this expression construct **(** **Figure 2B** **).** In addition to the immunofluorescence read-out system, which demonstrated a restored intracellular signaling, we also assessed whether the E-cadherin construct re-activates calcium-dependent cell-cell adhesion. E-cadherin-deficient cells transfected with Ecad-EGFP showed an increased cell aggregation **(** **Figure 2C** **)**. Notably, this effect was abrogated by the calcium chelating agent EDTA, arguing for the correct functionality of this E-cadherin expression construct **(** **Figure 2C** **)**.

### High Frequency of BCL2-negative Cases in Human Primary ILBCs

**Figure 3:**
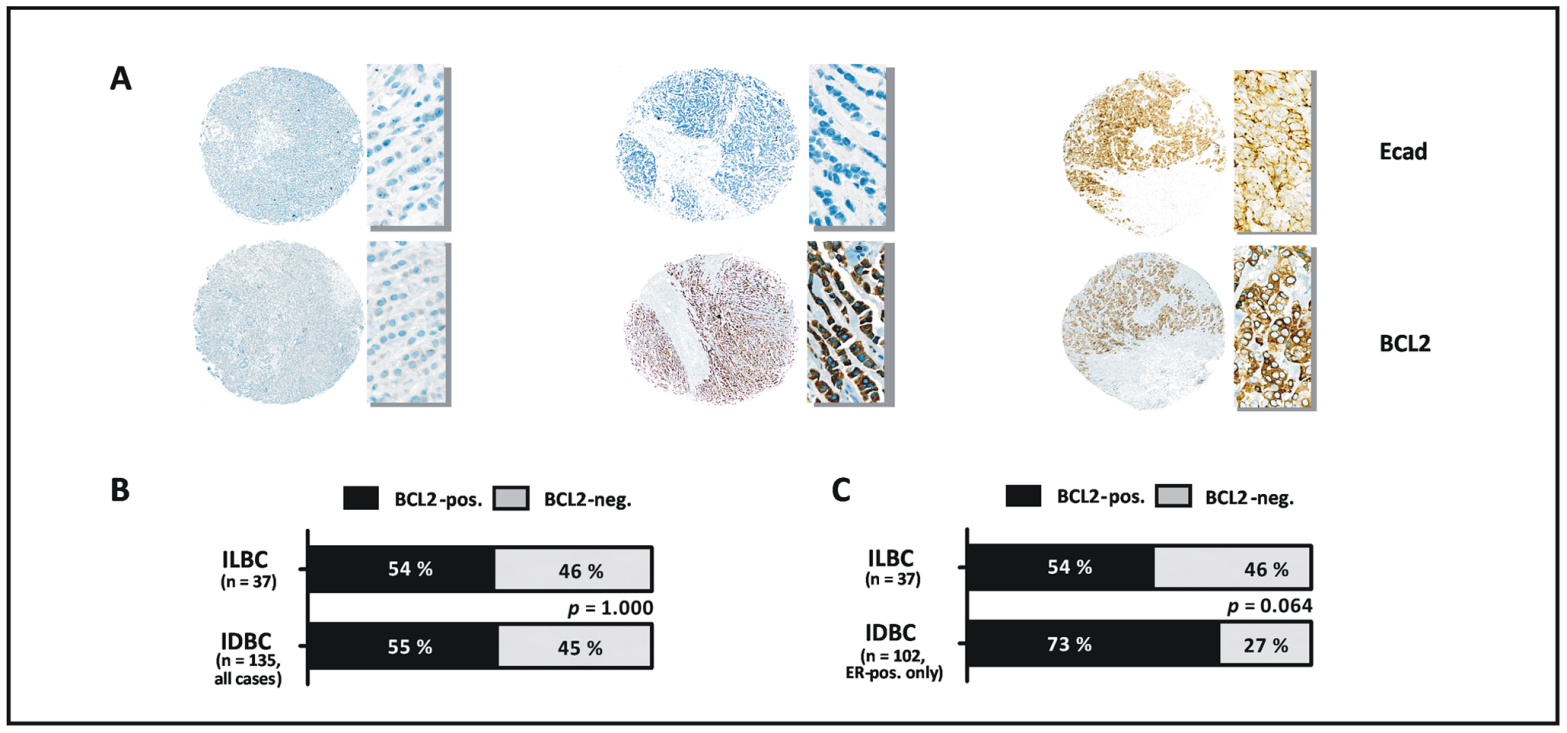
Primary ILBCs lack E-cadherin expression and are frequently BCL2-negative. (**A**) Representative immunohistochemical stainings of two primary ILBCs (left, middle) and one primary IDBC (right) for E-cadherin (Ecad) and BCL2, as performed on TMAs. An overview of the tumor cores embedded in the TMAs is shown on the left side of each cases and a detail photomicrograph (magnification, ×400) is shown on the right side of each case. (**B**) Comparison of the frequency of BCL2-positive/−negative cases in ILBCs versus IDBCs. Statistical significance was determined with Fisher’s exact test. (**C**) Comparison of BCL2 positive/−negative cases in ILBCs *versus* ER-positive IDBCs.

Reconstitution of E-cadherin in human ILBC cells *in vitro* failed to substantiate a reciprocal regulation between E-cadherin and BCL2. For several reasons, which are discussed below, this does not necessarily rule out such a regulatory interrelationship in human tumors *in vivo*. The human mammary epithelium is constitutively positive for BCL2-expression [40]. In breast cancer, loss of BCL2 has repeatedly been associated with high grade tumors and other aggressive carcinoma entities, which shows that BCL2 acts as a differentiation marker in the mammary gland [41], [42], [43]. In view of the recently proposed concept, that loss of E-cadherin propels malignant transformation by upregulation of BCL2, we hypothesized that primary ILBCs, which typically lack E-cadherin, might preferentially be BCL2-positive or retain BCL2 expression. Accordingly, BCL2 and E-cadherin were evaluated in a series of n = 172 human primary invasive breast cancers compiled on TMAs using immunohistochemical stainings. Consistent with previous findings [41], [42], [43], loss of BCL2 expression was associated with high histological grade, lack of ER, lack of PR, and high Ki67 labeling index (*P<*0.001, each) **(Table S1)**. ILBCs were almost uniformly E-cadherin-negative (97%), while IDBCs were almost exclusively E-cadherin-positive (96%) **(** **Table 2** **and** **Figure 3A** **)**. Despite this difference in E-cadherin expression in ILBCs *versus* IDBCs, the proportion of BCL2-positive/-negative cases was similar in the two entities (*P = *1.000) **(** **Figure 3B** **)**. Variation of the IRS cutoff defining BCL2-positive/-negative cases yielded similar results (data not shown). As a matter of fact, a large fraction of ILBCs (46%) showed essentially no BCL2 immunoreactivity **(** **Figure 3A and B** **)**. As ILBCs are typically ER-positive, we decided to restrict our tumor series to ER-positive cases for a refined analysis. A total of n = 139 ER-positive carcinomas entered this refined evaluation and these cases included all ILBCs **(Table S2 and S3)**. In this more focused tumor series, ILBCs even showed a trend towards a higher frequency of BCL2-negative cases (*P = *0.064) **(** **Figure 1C** **)**. Hence, primary ILBCs were almost uniformly E-cadherin-negative, but showed a comparatively high frequency of BCL2-negative cases, which indicates that BCL2 expression is dispensable during the development of these tumors.

### Similar or Reduced BCL2 Expression in LCIS Compared with the Adjacent Epithelium

**Figure 4:**
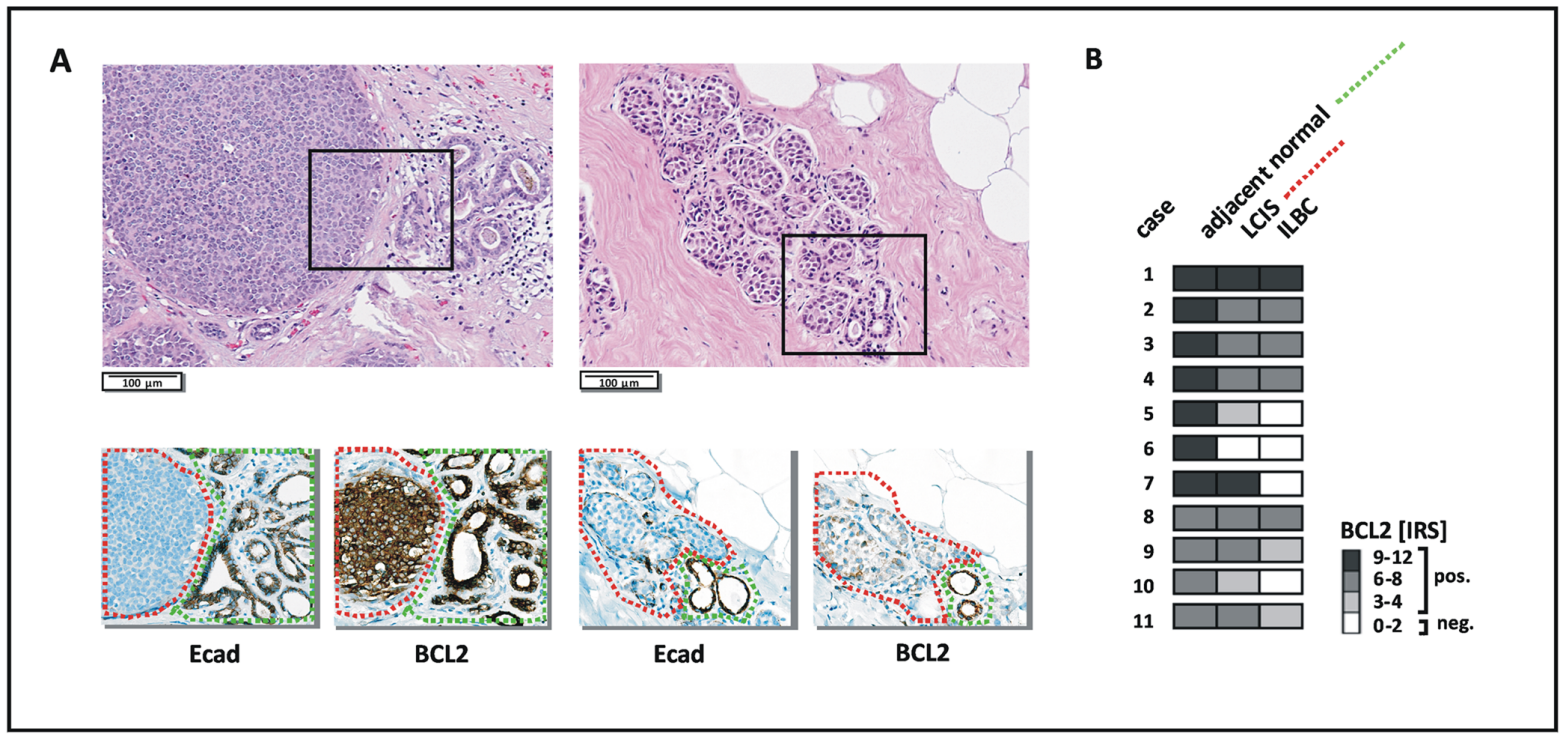
Similar or reduced BCL2 immunoreactivity in LCIS compared with the adjacent mammary epithelium. (**A**) Representative photomicrographs showing two LCIS lesions characterized by either similar (left, case #1) or reduced (right, case #10) BCL2 immunoreactivity compared with the adjacent E-cadherin-positive epithelium. HE stained sections are shown on top. Serial sections subjected to immunohistochemical staining of E-cadherin (Ecad) and BCL2 are shown below. (**B**) Overview on BCL2 immunoreactivity in LCIS lesions of n = 11 patients. At least three TDLUs affected by LCIS were considered per case, but showed essentially the same staining characteristics (not shown). A color scale indicative of the IRS is included on the right side.

Analysis of gene expression in tumor cohorts is an indirect way to assess potential regulatory interrelationships. Relevant associations may be obscured by inter-individual tumor heterogeneity. To circumvent this methodological limitation and to rule out the possibility that upregulation of BCL2 is restricted to the pre-invasive stage of tumor development driven by *CDH1* inactivation, we evaluated E-cadherin and BCL2 immunoreactivity in LCIS lesions. This provides the opportunity to use the normal mammary epithelium of affected TDLUs as an intra-individual reference for baseline expression of the gene of interest [15]. From the ILBCs represented in the TMA series, we selected n = 11 cases with synchronous LCIS lesions on separate FFPE tissue blocks for analysis of pre-invasive tumor cells. In line with previous findings [42], the normal mammary epithelium was always E-cadherin-positive and BCL2-positive **(** **Figure 4A** **)**. Also, there was essentially no variation in BCL2 immunoreactivity between different TDLUs within the same patient (data not shown). However, the E-cadherin-negative, neoplastic cellular component of TDLUs affected by LCIS showed a similar, a reduced or a complete loss of BCL2-immunoreactivity, when compared with the adjacent normal epithelium **(** **Figure 4A and B** **)**. No increased immunoreactivity for BCL2 was observed in any of the LCIS lesions evaluated **(** **Figure 4B** **)**.

## Discussion

Loss of E-cadherin can initiate tumor development, but the molecular mechanisms that mediate this tumor suppressive function are less well understood than the role of E-cadherin in cancer progression and metastasis [5],[6]. The recently described reciprocal regulation between E-cadherin and the anti-apoptotic oncoprotein BCL2, which is supposed to be mediated by Notch-1, provides an attractive new explanation how *CDH1* inactivation might propel neoplastic transformation [8]. According to this new model, loss of E-cadherin triggers an upregulation of Notch-1 expression and activity, which in turn induces increased BCL2 expression. BCL2 for its part is believed to increase cell survival in E-cadherin-deficient tumor cells [8]. Conversely, reconstitution of E-cadherin has been reported to counteract overexpression of BCL2 [8]. However, the relevance of this new model for human tumor development has remained uncertain, as previous studies have only focused on a limited number of *in vitro* cell models and few clinical tumor specimens [8]. ILBC is a special breast cancer entity associated with mutational *CDH1* inactivation and loss of E-cadherin expression [2], [12], [13], [14], [15], [16], [17]. Using ILBC cell lines, primary tumors and pre-invasive lesions as a model, the present work aimed to determine whether reciprocal upregulation of BCL2 is an important determinant of tumor development driven by inactivation of *CDH1*/E-cadherin.

Characterization of human ILBC cell lines harboring deleterious *CDH1* mutations revealed different levels of BCL2 expression. MDA-MB-134 and IPH-926 ILBC cells expressed little or no BCL2, while SUM-44-PE cells were BCL2-positive. Contrary to previous findings in MDA-MB-435/M14 [8], reconstitution of full-length wild-type E-cadherin failed to downregulate BCL2 in SUM-44-PE cells. However, we could also not confirm a downregulation of BCL2 in MDA-MB-435/M14 cells, which had been used in the original study describing the reciprocal regulation between E-cadherin and BCL2 [8]. Moreover, Notch-1 protein expression was not detectable in any of the E-cadherin-deficient ILBC cell lines tested, arguing against the existence of a regulatory axis between E-cadherin, Notch-1 and BCL2.

However, one might insist that the E-cadherin expression construct used in the present work could have had an impaired functionality due to the EGFP-tag, which is required for pre-analytical enrichment of E-cadherin-positive cells. Yet, proper function of the Ecad-EGFP construct had been verified by several assays devised to read out restored intracellular signaling (relocation of p120-catenin and Kaiso to the cell membrane or nucleus) and restored calcium dependent cell-cell adhesion (cell aggregation assay). Alternatively, one might consider that the regulatory axis between E-cadherin and BCL2 has become fixed in our MDA-MB-435/M14, SUM-44-PE and BT-549 cells due to the long time since their establishment **(** **Table 1** **)**. Even so, the absence of a significant overexpression of BCL2 and Notch-1 in the E-cadherin-deficient MDA-MB-134 and IPH-926 ILBC cells is not in line with the concept that loss of E-cadherin triggers an upregulation of BCL2 through increased expression and activity of Notch-1.

Immunohistochemical analysis of human primary breast cancers revealed that a comparatively large proportion of ILBCs lack BCL2 expression, despite complete loss of E-cadherin. This is consistent with a previous study reported by Coradini *et al.* [44]. When compared with an appropriate control group of ER-positive ductal carcinomas, ILBCs even showed a trend towards a higher frequency of BCL2-negative cases. This is not in line with the proposed reciprocal regulation between E-cadherin and BCL2. In fact, this observation strongly suggests that BCL2 is dispensable during the development of E-cadherin-deficient ILBC. In line with this assumption, evaluation of LCIS lesions demonstrated that E-cadherin-negative pre-invasive tumor cells rather exhibit a reduced, but not an increased, BCL2 immunoreactivity, when compared with the adjacent normal epithelium. This also argues against an upregulation of BCL2 at the site of *CDH1* inactivation within the human mammary epithelium. Interestingly, however, we observed retained BCL2 expression in 4 out of 5 IDBCs with abrogated E-cadherin expression. E-cadherin-negative IDBC is a very uncommon tumor phenotype and, possibly due to the extremely small sample size, also lacked a significant association with the BCL2 status. There could be a functional interrelationship between E-cadherin and BCL2 in the extremely rare E-cadherin-negative IDBCs, but apparently not in the much more common E-cadherin-negative ILBCs.

Taken together, upregulation of BCL2 is not effective in human ILBC cell lines, primary tumors and pre-invasive lesion. As ILBC is the key tumor entity associated with loss of E-cadherin expression, this suggests that reciprocal regulation of BCL2 is not a major mechanism of tumor development driven by *CDH1* inactivation.

## Supporting Information

Figure S1 **Notch-1 protein expression, as detected by Western blot.** Pos cntrl; positive control (293T cells transfected with Notch-1).
(TIF)

Table S1 **Relationship between BCL2 status and clinicopathological factors.**
(DOC)

Table S2 **Characteristics of primary tumors, ER-pos. subset.**
(DOC)

Table S3 **Relationship between BCL2 status and clinicopathological factors, ER-pos. subset.**
(DOC)
