# Supplementary figures and images for: Is Upregulation of BCL2 a Determinant of Tumor Development Driven by Inactivation of CDH1/E-Cadherin?
Source: PLoS One. 2013 Aug 30;8(8):e73062. doi: 10.1371/journal.pone.0073062 (PMC3758309; doi:10.1371/journal.pone.0073062)

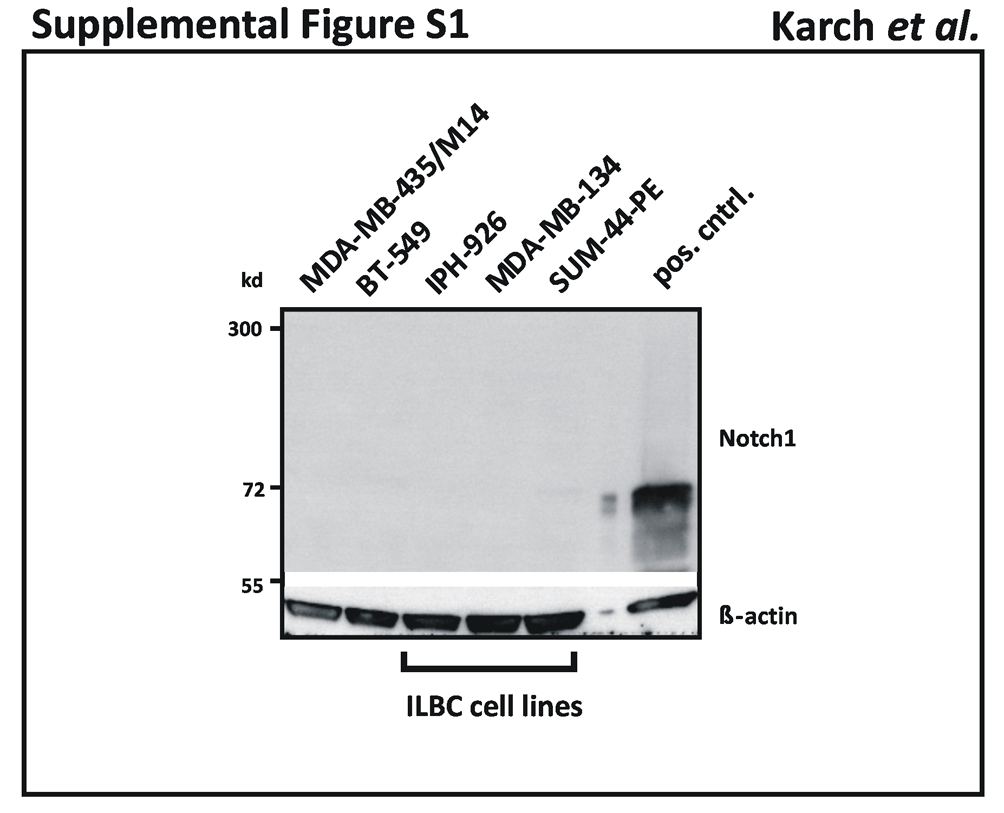

Supplement: Figure S1 — Notch-1 protein expression, as detected by Western blot. Pos cntrl; positive control (293T cells transfected with Notch-1). (TIF) [file pone.0073062.s001.tif]
