# Supplementary material for: Is Upregulation of BCL2 a Determinant of Tumor Development Driven by Inactivation of CDH1/E-Cadherin?
Source: PLoS One. 2013 Aug 30;8(8):e73062. doi: 10.1371/journal.pone.0073062 (PMC3758309; doi:10.1371/journal.pone.0073062)
Supplement: Table S1 — Relationship between BCL2 status and clinicopathological factors. (DOC) [file pone.0073062.s002.doc]

|  | Supplemental Data Table S1 | | | | | | |  |
| --- | --- | --- | --- | --- | --- | --- | --- | --- |
|  | **Relationship between BCL2 status and clinicopathological factors** | | | | | | |  |
|  |  |  | **BCL2- negative** | | **BCL2- positive** | | ***p* value** |  |
|  | ***cases*** |  | 78 | (46) | 94 | (55) |  |  |
|  | ***age*** |  |  |  |  |  | *p* = 1.000^1^ |  |
|  | >60 |  | 33 | (46) | 39 | (54) |  |  |
|  | <60 |  | 45 | (45) | 55 | (55) |  |  |
|  | ***histological type*** |  |  |  |  |  | *p* = 1.000^1^ |  |
|  | ILBC |  | 17 | (46) | 20 | (54) |  |  |
|  | IDBC |  | 61 | (45) | 74 | (55) |  |  |
|  | ***pT status*** |  |  |  |  |  | *p* = 0.069^1^ |  |
|  | pT1/ pT2 |  | 63 | (42) | 86 | (58) |  |  |
|  | pT3/ pT4 |  | 14 | (64) | 8 | (36) |  |  |
|  | ***pN status*** |  |  |  |  |  | *p* = 0.129^1^ |  |
|  | pN0 |  | 38 | (40) | 56 | (60) |  |  |
|  | pN1+ |  | 31 | (54) | 26 | (46) |  |  |
|  | ***histological grade*** |  |  |  |  |  | *p* < 0.001^2^ |  |
|  | G1 |  | 1 | (7) | 14 | (93) |  |  |
|  | G2 |  | 34 | (35) | 63 | (65) |  |  |
|  | G3 |  | 43 | (72) | 17 | (28) |  |  |
|  | ***estrogen receptor*** |  |  |  |  |  | *p* < 0.001^1^ |  |
|  | positive |  | 45 | (32) | 94 | (68) |  |  |
|  | negative |  | 33 | (100) | 0 | (0) |  |  |
|  | ***progesterone receptor*** |  |  |  |  |  | *p* < 0.001^1^ |  |
|  | positive |  | 30 | (30) | 70 | (70) |  |  |
|  | negative |  | 48 | (67) | 24 | (33) |  |  |
|  | ***c-erbB2 expression*** |  |  |  |  |  | *p* = 0.010^2^ |  |
|  | 0, 1+ |  | 69 | (43) | 92 | (57) |  |  |
|  | 2+ |  | 2 | (67) | 1 | (33) |  |  |
|  | 3+ |  | 7 | (88) | 1 | (12) |  |  |
|  | ***E-cadherin (in ILBC)*** |  |  |  |  |  | *p* = 1.000^1^ |  |
|  | positive |  | 0 | (0) | 1 | (100) |  |  |
|  | negative |  | 17 | (47) | 19 | (53) |  |  |
|  | ***E-cadherin (in IDBC)*** |  |  |  |  |  | *p* = 0.378^1^ |  |
|  | positive |  | 60 | (46) | 70 | (54) |  |  |
|  | negative |  | 1 | (20) | 4 | (80) |  |  |
|  | ***Ki67 LI*** |  |  |  |  |  | *p* < 0.001^2^ |  |
|  | <10 |  | 5 | (17) | 25 | (83) |  |  |
|  | >10, <24 |  | 33 | (37) | 56 | (63) |  |  |
|  | >25 |  | 40 | (75) | 13 | (25) |  |  |
|  |  |  |  |  |  |  |  |  |

Numbers in parentheses indicate percentages

^1^Fisher's exact test

^2^Chi square test for trends
