# Supplementary material for: Is Upregulation of BCL2 a Determinant of Tumor Development Driven by Inactivation of CDH1/E-Cadherin?
Source: PLoS One. 2013 Aug 30;8(8):e73062. doi: 10.1371/journal.pone.0073062 (PMC3758309; doi:10.1371/journal.pone.0073062)
Supplement: Table S2 — Characteristics of primary tumors, ER-pos. subset. (DOC) [file pone.0073062.s003.doc]

|  | **Supplemental Data Table S2** | | |  |  |
| --- | --- | --- | --- | --- | --- |
|  | Characteristics of primary tumors, ER-pos. subset | | | |  |
|  |  |  | **number of cases** | **percent** |  |
|  | ***cases*** |  | 139 | 100 |  |
|  | ***age*** |  |  |  |  |
|  | >60 |  | 56 | 40 |  |
|  | <60 |  | 83 | 60 |  |
|  | ***histological type*** |  |  |  |  |
|  | ILBC |  | 37 | 27 |  |
|  | IDBC |  | 102 | 73 |  |
|  | ***pT status*** |  |  |  |  |
|  | pT1/ pT2 |  | 120 | 86 |  |
|  | pT3/ pT4 |  | 18 | 13 |  |
|  | pTx |  | 1 | 1 |  |
|  | ***pN status*** |  |  |  |  |
|  | pN0 |  | 77 | 55 |  |
|  | pN1+ |  | 43 | 31 |  |
|  | pNx |  | 19 | 14 |  |
|  | ***histological grade*** |  |  |  |  |
|  | G1 |  | 15 | 11 |  |
|  | G2 |  | 90 | 65 |  |
|  | G3 |  | 34 | 24 |  |
|  | ***estrogen receptor*** |  |  |  |  |
|  | positive |  | 139 | 100 |  |
|  | negative |  | 0 | 0 |  |
|  | ***progesterone receptor*** |  |  |  |  |
|  | positive |  | 100 | 72 |  |
|  | negative |  | 39 | 28 |  |
|  | ***c-erbB2 expression*** |  |  |  |  |
|  | 0, 1+ |  | 132 | 95 |  |
|  | 2+ |  | 3 | 2 |  |
|  | 3+ |  | 4 | 3 |  |
|  | ***E-cadherin (in ILBC)*** |  |  |  |  |
|  | positive |  | 1 | 3 |  |
|  | negative |  | 36 | 97 |  |
|  | ***E-cadherin (in IDBC)*** |  |  |  |  |
|  | positive |  | 98 | 96 |  |
|  | negative |  | 4 | 4 |  |
|  | ***Ki67 LI*** |  |  |  |  |
|  | <10 |  | 30 | 22 |  |
|  | >10, <24 |  | 83 | 60 |  |
|  | >25 |  | 26 | 18 |  |
|  |  |  |  |  |  |
