# Supplementary material for: Is Upregulation of BCL2 a Determinant of Tumor Development Driven by Inactivation of CDH1/E-Cadherin?
Source: PLoS One. 2013 Aug 30;8(8):e73062. doi: 10.1371/journal.pone.0073062 (PMC3758309; doi:10.1371/journal.pone.0073062)
Supplement: Table S3 — Relationship between BCL2 status and clinicopathological factors, ER-pos. subset. (DOC) [file pone.0073062.s004.doc]

|  | Supplemental Data Table S3 | | | | | | |  |
| --- | --- | --- | --- | --- | --- | --- | --- | --- |
|  | **Relationship between BCL2 status and clinicopathological factors, ER-pos. subset** | | | | | | |  |
|  |  |  | **BCL2- negative** | | **BCL2- positive** | | ***p* value** |  |
|  | ***cases*** |  | 45 | (32) | 94 | (68) |  |  |
|  | ***age*** |  |  |  |  |  | *p =*0.715^1^ |  |
|  | >60 |  | 17 | (30) | 39 | (70) |  |  |
|  | <60 |  | 28 | (34) | 55 | (66) |  |  |
|  | ***histological type*** |  |  |  |  |  | *p =*0.064^1^ |  |
|  | ILBC |  | 17 | (46) | 20 | (54) |  |  |
|  | IDBC |  | 28 | (27) | 74 | (73) |  |  |
|  | ***pT status*** |  |  |  |  |  | *p =*0.030^1^ |  |
|  | pT1/ pT2 |  | 34 | (28) | 86 | (72) |  |  |
|  | pT3/ pT4 |  | 10 | (56) | 8 | (44) |  |  |
|  | ***pN status*** |  |  |  |  |  | *p =*0.220^1^ |  |
|  | pN0 |  | 21 | (27) | 56 | (72) |  |  |
|  | pN1+ |  | 17 | (40) | 26 | (60) |  |  |
|  | ***histological grade*** |  |  |  |  |  | *p =*0.002^2^ |  |
|  | G1 |  | 1 | (7) | 14 | (93) |  |  |
|  | G2 |  | 27 | (30) | 63 | (70) |  |  |
|  | G3 |  | 17 | (50) | 17 | (50) |  |  |
|  | ***progesterone receptor*** |  |  |  |  |  | *p =*0.420^1^ |  |
|  | positive |  | 30 | (30) | 70 | (70) |  |  |
|  | negative |  | 15 | (38) | 24 | (62) |  |  |
|  | ***c-erbB2 expression*** |  |  |  |  |  | *p =*0.026^2^ |  |
|  | 0,1+ |  | 40 | (30) | 92 | (70) |  |  |
|  | 2+ |  | 2 | (67) | 1 | (33) |  |  |
|  | 3+ |  | 3 | (75) | 1 | (25) |  |  |
|  | ***E-cadherin (in ILBC)*** |  |  |  |  |  | *p =*1.000^1^ |  |
|  | positive |  | 0 | (0) | 1 | (100) |  |  |
|  | negative |  | 17 | (47) | 19 | (53) |  |  |
|  | ***E-cadherin (in IDBC)*** |  |  |  |  |  | *p*= 0.573^1^ |  |
|  | positive |  | 28 | (29) | 70 | (71) |  |  |
|  | negative |  | 0 | (0) | 4 | (100) |  |  |
|  | ***Ki67 LI*** |  |  |  |  |  | *p =*0.008^2^ |  |
|  | <10 |  | 5 | (17) | 25 | (83) |  |  |
|  | >10, <24 |  | 27 | (33) | 56 | (67) |  |  |
|  | >25 |  | 13 | (50) | 13 | (50) |  |  |
|  |  |  |  |  |  |  |  |  |

Numbers in parentheses indicate percentages

^1^Fisher's exact test

^2^Chi square test for trends
